# Comment on “Effectiveness of a Group B outer membrane vesicle meningococcal vaccine in preventing hospitalization from gonorrhea in New Zealand: a retrospective cohort study, *Vaccines*, 2019, 1, 5; doi:10.3390/vaccines7010005”

**DOI:** 10.3390/vaccines7010031

**Published:** 2019-03-14

**Authors:** Chris Kenyon

**Affiliations:** 1 HIV/STI Unit, Institute of Tropical Medicine, 2000 Antwerp, Belgium; ckenyon@itg.be; Tel.: +32-3248-0796; 2 Division of Infectious Diseases and HIV Medicine, University of Cape Town, Anzio Road, Observatory 7700, South Africa

**Keywords:** *N. gonorrhoea*, vaccine efficacy, gonorrhoea, MeNZB, meningitis B vaccine

## Abstract

Available evidence suggests MeNZB™ is not associated with a durable effect against *N. gonorrhoeae*.

Even a partially effective vaccine against *N. gonorrhoeae* could be of considerable utility to sexually transmitted infection (STI) control efforts. In their retrospective cohort study, Paynter et al., found that meningococcal B vaccination (MeNZB™) was associated with a vaccine effectiveness (VE) of 24% against hospitalization caused by gonorrhoea [1]. They concluded, based on this study and a previous case control study in the same population that showed a similar VE [2], that this type of vaccine may offer a durable option to control *N. gonorrhoeae*. Whilst we wish this were true, we consider it important to note that in both studies the VE declined with time. In the case control study, VE declined from a statistically significant 31% to a non-significant 9% after 5 years [2]. Likewise, in the current study, and as noted by the authors, there was no significant VE in the youngest of three age groups vaccinated (median age 8). This cohort would only have been exposed to *N. gonorrhoeae* in later years when they became sexually active [1]. These findings are compatible with at least two explanations. Firstly, this could be due to the waning efficacy of meningitis B vaccines over time that has been noted in other studies and may occur within 6 months of vaccination [3,4]. Secondly, a hallmark of *N. gonorrhoeae* is its ability to adapt to selection pressures. Included in its evolutionary toolbox is a highly developed system of transformation that enables it to take up DNA sequences from other microbes and thereby adapt to circumvent adverse selection pressures [5,6]. This, along with other mechanisms, have enabled it to evolve resistance to every antimicrobial class that has been used against it [5]. Resistance evolves in 2–4 weeks in vitro and in around 3 years in clinical practice [7]. Phylogenetic and/or in vitro studies have established that the gonococcus has used these mechanisms to take up resistance conferring DNA from a range of organisms including numerous commensal *Neisseria* spp. [8,9]. Future studies assessing the VE of meningitis B vaccines against *N. gonorrhoeae* could test this hypothesis by assessing if vaccination selects for changes in vaccine targets in *N. gonorrhoeae*. Until further studies have established a durable VE of MeNZB™ against *N. gonorrhoeae* we consider it prudent to interpret the two studies referred to above as demonstrating an initial moderate VE but little or no long term VE.
